# Rectoperineal Fistula Presented 5 Months After Repair of Severe Obstetric Perineal Laceration: A Case Report

**DOI:** 10.3389/fsurg.2021.637719

**Published:** 2021-06-24

**Authors:** Yusuke Ohara, Tsuyoshi Enomoto, Yohei Owada, Katsuji Hisakura, Yoshimasa Akashi, Koichi Ogawa, Manami Doi, Kazuhiro Takahashi, Osamu Shimomura, Kinji Furuya, Jaejeong Kim, Shinji Hashimoto, Rena Ohara, Mana Obata-Yasuoka, Hiromi Hamada, Tatsuya Oda

**Affiliations:** ^1^Department of Gastrointestinal and Hepato-Biliary-Pancreatic Surgery, Faculty of Medicine, University of Tsukuba, Tsukuba, Japan; ^2^Department of Obstetrics and Gynecology, Faculty of Medicine, University of Tsukuba, Tsukuba, Japan

**Keywords:** rectoperineal fistula, obstetric laceration, anal incontinence, surgical site infection, gastrointestinal surgery

## Abstract

**Introduction:** Obstetric severe perineal laceration can frequently occur as a surgical site infection (SSI), which sometimes leads to rectovaginal fistula after repair. We encountered a rare case of a rectoperineal fistula 5 months after repair of a severe perineal laceration.

**Case presentation:** The patient was a 39-year-old woman who underwent repair of a fourth-degree perineal laceration after vaginal delivery. Five months after primary repair, she presented with perineal swelling and pain followed by uncontrollable flatulence or passage of feces at the perineum, which was finally diagnosed as a rectoperineal fistula. Transperineal repair with fistulous tract excision was performed for the rectoperineal fistula. Closure of the rectum, perineal body, and vagina was performed layer-by-layer constructing a thick perineum to prevent anal dysfunction. The fistula was successfully closed, and the patient did not show any symptoms of fecal incontinence 6 months after surgery.

**Discussion:** As the rectoperineal fistula might have resulted in SSI at the primary repair of the obstetric injury, the delayed occurrence of the rectoperineal fistula was unusual. A perineal approach should be performed for complete fistulous tract excision, reconstruction of a robust perineal structure, and preservation of anal sphincter function.

## Introduction

Obstetric perineal lacerations, as 53-79% of women sustain them, are often accompanied by vaginal delivery (1, 2). Traditionally, obstetric perineal laceration is classified into four degrees according to the anatomical structure involved (3). Third-degree laceration extends to the anal external and/or internal sphincter. Fourth-degree laceration extends to the rectal mucosa. These severe lacerations with injury to the anal sphincter occurs in 6.4% of women (4), leading to fecal incontinence. Careful recognition and adequate repair of lacerations are essential for preventing postpartum anal incontinence.

The purpose of repairing a third- or fourth-degree laceration is to restore the continuity of both the external and internal anal sphincters (5). Moreover, a thick perineal body and rectovaginal septum should be constructed to provide muscular and structural support in the thin area between the anterior anorectal region and vagina. Multilayer repair is optimal for severe laceration, maintaining the strength of perianal tissue, and preserving anal continence (3).

The repair of these severe lacerations carries a potential risk of surgical site infection (SSI), as the wound is easily contaminated by intestinal or epidermal bacteria. Goldaber et al. reported that 3.6% of patients with fourth-degree lacerations developed SSI after the repair (6). Venkatesh et al. documented that 101 of 2,500 women who underwent vaginal delivery presented with anorectal complications (7). They reported that 25 patients had a rectovaginal fistula; in contrast, only two patients (0.08%) had an anoperineal fistula. Here, we report a rare case of delayed rectoperineal fistula 5 months after repair of a fourth-degree perineal laceration resulting from a vaginal delivery.

## Case Presentation

A 39-year-old woman with a history of cesarean section for first childbirth was admitted to the University of Tsukuba Hospital for a second pregnancy. At 40 weeks of gestation for postdate pregnancy, a left lateral episiotomy followed by a forceps delivery was performed, and a baby weighing 3.93 kg was born in good condition. A fourth-degree perineal laceration was found on perineal examination immediately after delivery. Briefly, a vaginal examination showed a 7 cm laceration in the episiotomy. Rectal examination showed a 3 cm laceration in the lower rectum, but it did not advance to the anal ring. The external and internal sphincter injuries were minor.

The first operation for perineal laceration was performed under spinal anesthesia in the lithotomy position on the day of delivery. A multilayer closure was performed followed by independent sutures of the rectal wall, musculature of the perineal body with sphincter, vaginal epithelium, and skin. All the sutures were interrupted stitches using 4-0 monofilament absorbable sutures. Antibiotics (cefmetazole) were administered prior to the surgery and used until 5 days after surgery. Wound dehiscence was not observed, and the patient was discharged 11 days after surgery. The patient did not show any symptoms of fecal incontinence, SSI, or rectovaginal fistula on the outpatient examination 1 month after surgery.

**Figure 1:**
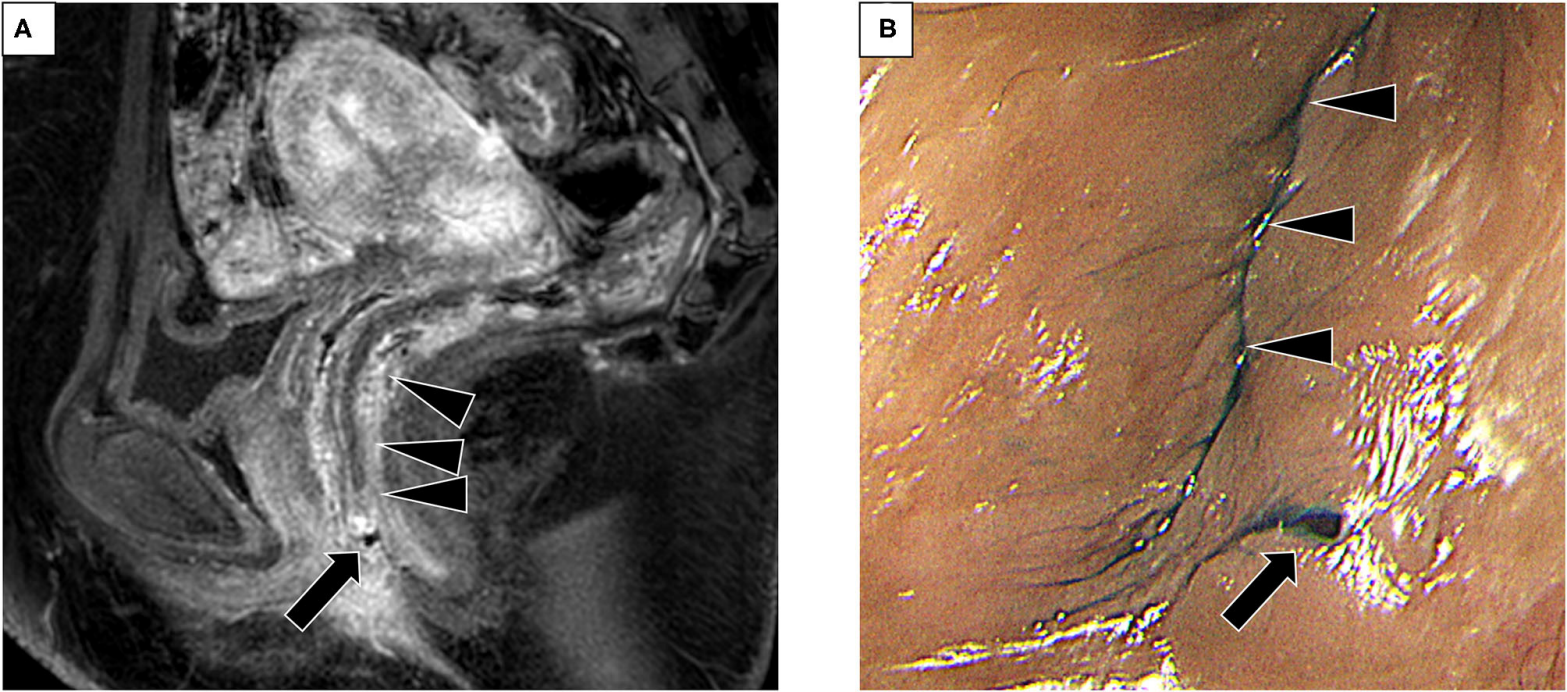
Pre-operative examination for the diagnosis of rectoperineal fistula **(A)** Magnetic resonance imaging (sagittal view) showing a small tract from the lower rectum. Arrow, small tract. Arrowhead, rectum. **(B)** Small fistula on the perineal skin visualized after insertion of indigo carmine poured into the rectum (arrow). Arrowhead, vaginal orifice.

**Figure 2:**
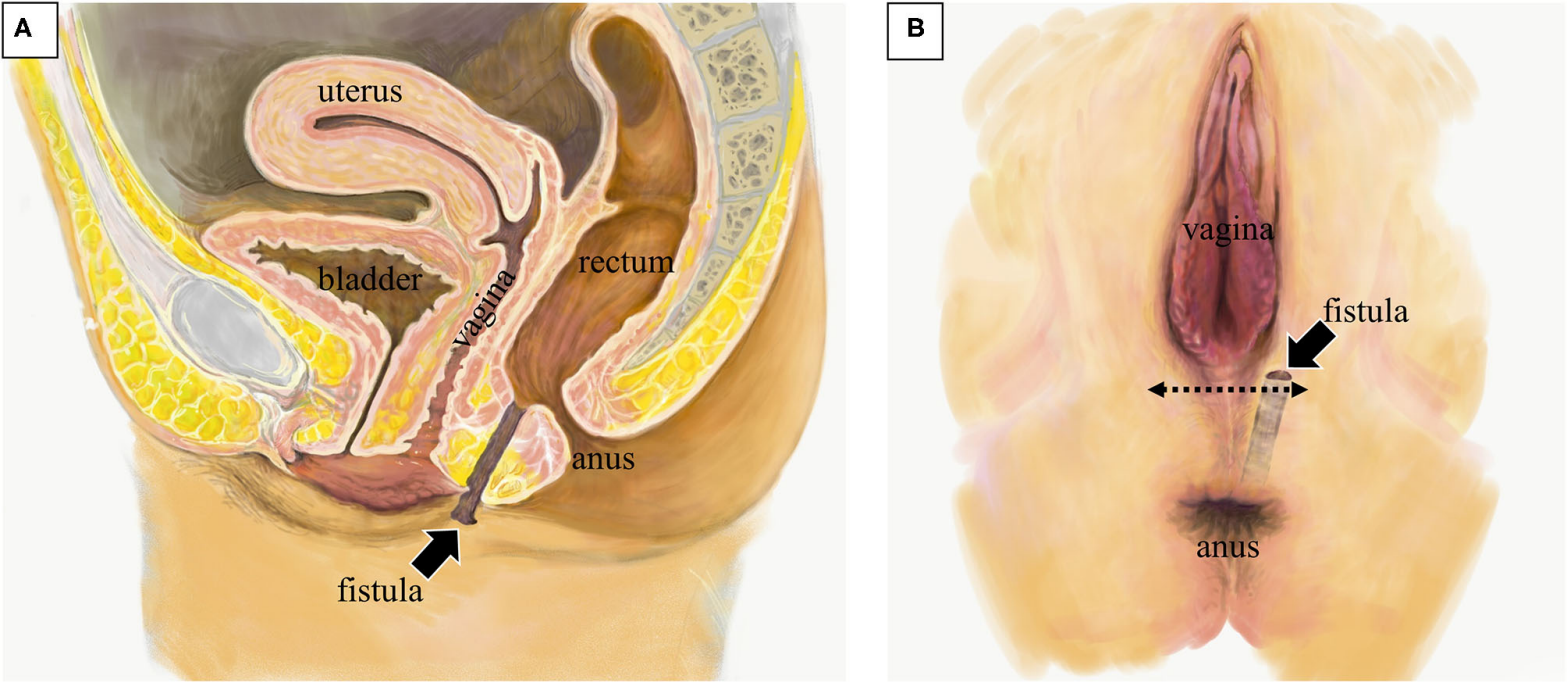
Anatomical schema of the rectoperineal fistula. **(A)** Sagittal image showing the position of fistula tract (arrow). **(B)** Image of perineal surface. The fistula opened nearby the vaginal orifice. Perineal approach with transverse incision was placed at the surgery (dotted arrow).

Five months after the first surgery, the patient noticed perineal swelling and pain, followed by an uncontrollable passage of gas or feces at the perineum. A small pinhole with a discharge was noted at the left perineum, at the 5-O'clock direction of the vagina, and 5 mm from the vaginal orifice. Magnetic resonance imaging revealed a small fistula tract from the lower rectum, but an external opening or perianal abscess was not revealed (Figure 1A). Colonoscopy also showed a small orifice at the anterior wall of the lower rectum (above the dentate line), and indigo carmine poured into the rectum was discharged from the perineal opening (Figure 1B). Vaginoscopy did not reveal a fistula tract in the vagina. Finally, a rectoperineal fistula was diagnosed. No evidence of Crohn's disease was observed. Anatomical schema demonstrating the position of fistula tract was shown in Figure 2.

**Figure 3:**
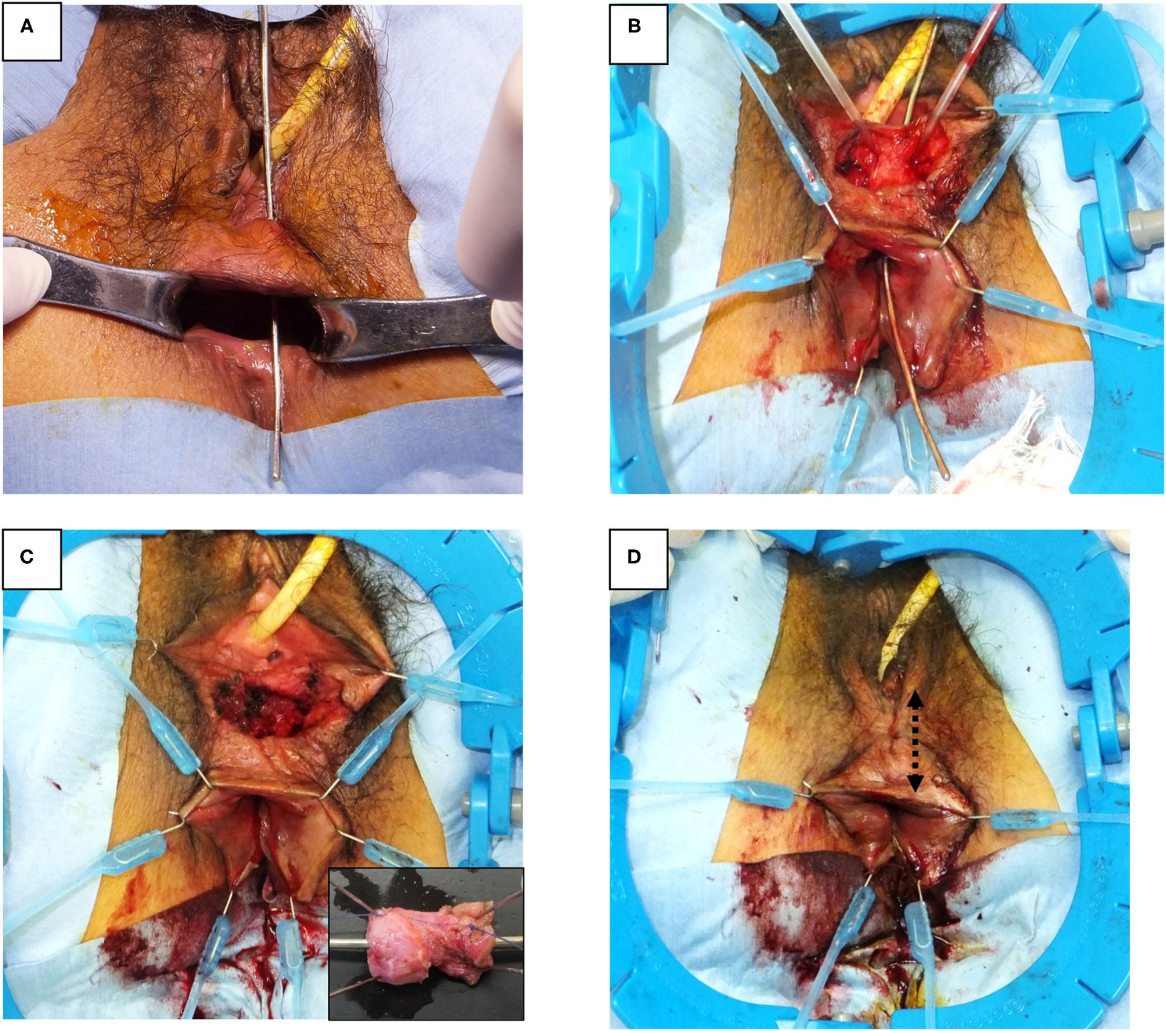
Surgical procedure of rectoperineal fistula repair. **(A)** Identification of rectoperineal fistula with a probe through the fistula tract. **(B)** Transverse perineal incision and dissection of rectovaginal septum exposing fistula tract. **(C)** Fistula tract is completely excised from the rectovaginal septum. Small box, macroscopic feature of fistula tract (2 × 1 cm). **(D)** Layered closure of rectum, perineal body, vagina, and skin are performed, respectively. The skin was closed along vertical direction (dotted arrow).

Seven months after the first operation, a second operation, transperineal repair for rectoperineal fistula was performed (Figures 2B, 3). Briefly, the patient received general anesthesia in the lithotomy position. A Lone Star Retractor System (CooperSurgical, Inc., Trumbull, USA) was attached to the anus and vagina. A 2 mm probe was advanced through the rectoperineal fistula to guide the route of the fistula. A 4 cm transverse incision was made on the perineal skin, 5 mm from the vaginal orifice. Dissection of the rectovaginal septum was performed widely, separating the vagina and rectum, and 2 cm of the entire fistulous tract was excised. A three-layer closure (rectum, perineal body, and vagina, respectively) was performed with interrupted sutures using 4-0 monofilament absorbable sutures. The subcutaneous tissue and skin were sutured horizontally; thus, the incision was changed in the vertical direction, keeping thick perineal tissue. A trans-anal drain was placed until 5 days after surgery. Antibiotics (flomoxef sodium) were administered prior to the surgery and used until 3 days after surgery. The patient was discharged 10 days after surgery. The patient did not show any symptoms of SSI, rectovaginal fistula, or fecal incontinence on the outpatient examination 6 months after surgery. Anorectal manometry showed a normal anal sphincter function. Colonoscopy showed no dip or fistula on the rectal mucosa.

## Discussion

Present case exhibited two unusual features. First, the fistula tract due to severe perineal laceration advanced to perineal skin, not to anorectum. Second, the rectoperineal fistula presented symptoms several months after initial repairment. These factors would remarkably affect surgical strategy and post-operative surveillance of severe obstetric lacerations.

Perineal wound complications after severe obstetric perineal lacerations are uncommon, but they can lead to significant morbidity including chronic pain, incontinence, loss of sexual function, and rectovaginal fistula (8, 9). Rectovaginal fistula after vaginal delivery can result from a failure to recognize the primary obstetric injury, inadequate repair, or surgical site infection of a repaired wound. Rectoperineal fistula, which might be similar to rectovaginal fistula, is a rare complication even after severe perineal lacerations. Lewicky-Gaupp et al. demonstrated the risk of wound complications in a prospective cohort that enrolled 268 women with obstetric anal sphincter injury. They showed that 53 (19.8%) women developed wound infection and 66 (24.6%) developed wound breakdown; however, only one woman (0.4%) developed a rectoperineal fistula.

The natural history of rectoperineal fistula after obstetric injuries has not been well-clarified; however, it might develop as an epithelialized track that connects the perineal skin and the rectum when a perianal abscess ruptures or is drained. As tight adhesion of the vaginal wall might block rectovaginal fistulation, the abscess might proceed toward the perineal skin, previously injured by an obstetric laceration or episiotomy. The timing of the occurrence of wound problems after obstetric injury varied among several reports. The rectoperineal fistula in the case of Lewicky-Gaupp et al. was presented and treated within 2 weeks of delivery (10). Stock et al. showed that the timing of readmission of wound complications after obstetric anal sphincter injury was 9 weeks after delivery at the latest (11). On the other hand, Barranger et al. presented three cases of anal fistula as a late complication of mediolateral episiotomy, which had been diagnosed 5, 9, and 24 months after delivery, respectively (12). The early timing of fistulation was caused by bacteria-contaminated at vaginal delivery; however, it must be noted that perianal abscess could be produced even at a later time after vaginal delivery.

A rectoperineal fistula should be repaired in consideration to the rectovaginal fistula. Rectovaginal fistula can be treated through abdominal, rectal, vaginal, perineal, trans-sphincteric, or trans-sacral approaches (13). For simple rectoperineal fistula, as in our case, a rectal or perineal approach followed by layer closure might be appropriate. Our perineal approach could easily recognize and dissect the fistulous tract, avoiding sphincteric injury. Due to the dissection of the rectovaginal septum, the posterior vaginal and anterior rectal wall are widely mobilized. Therefore, the layered closure of the rectal wall, perineal body, and vaginal wall could tightly construct the perineum without tissue tension. Wiskind and Thompson reported that all patients who underwent rectoperineal repair had satisfactory recovery with no recurrence in a series of 21 patients with rectovaginal fistula (14). Although the advancement flap through the rectal approach might be a procedure for rectoperineal fistula, it was not practiced in our case in which slight and fragile tissues remained between the vagina and rectum due to the primary obstetric injury. A thick perineal cushion was necessary to prevent recurrence or anal incontinence, which could not be created with an advancement flap. Hull et al. also reported that 78% of patients who underwent rectoperineal repair after obstetric injury was successfully repaired, providing better outcomes than the rectal advancement flap method (15).

## Conclusion

Severe obstetric perineal laceration can result in a vaginal or anorectal fistula even several months after primary repair, and the fistula may infrequently be opened at the perineal skin. Surgery for rectoperineal fistula requires complete fistulous tract excision under the distorted anatomy of the perineum and robust perineal reconstruction preventing recurrence and anal dysfunction.

## Data Availability Statement

The raw data supporting the conclusions of this article will be made available by the authors, without undue reservation.

## Ethics Statement

The studies involving human participants were reviewed and approved by University of Tsukuba. The patients/participants provided their written informed consent to participate in this study.

## Author Contributions

YOh, TE, and YOw performed surgical operation. KH, YA, KO, MD, KT, OS, KF, and SH planned and approved the surgical treatments. RO, MO-Y, and HH managed the obstetric treatment. JK supported the illustration in this article. YOh and TO were major contributors in writing the manuscript. All authors substantially contributed to the manuscript and read and approved the final manuscript.

## Conflict of Interest

The authors declare that the research was conducted in the absence of any commercial or financial relationships that could be construed as a potential conflict of interest.

## References

[1] Smith LA Price N Simonite V Burns EE. Incidence of and risk factors for perineal trauma: a prospective observational study. BMC Pregnancy Childbirth. (2013) 13:59. 10.1186/1471-2393-13-59 23497085 PMC3599825

[2] Rogers RG Leeman LM Borders N Qualls C Fullilove AM Teaf D. Contribution of the second stage of labour to pelvic floor dysfunction: a prospective cohort comparison of nulliparous women. BJOG. (2014) 121:1145–53; discussion 54. 10.1111/1471-0528.12571 24548705 PMC4565727

[3] Practice Bulletin No. 165: Prevention and Management of Obstetric Lacerations at Vaginal Delivery. Obstet Gynecol. (2016) 128:e115. 10.1097/AOG.0000000000001523 27333357

[4] Landy HJ Laughon SK Bailit JL Kominiarek MA Gonzalez-Quintero VH Ramirez M. Characteristics associated with severe perineal and cervical lacerations during vaginal delivery. Obstet Gynecol. (2011) 117:627–35. 10.1097/AOG.0b013e31820afaf2 21343766 PMC3132187

[5] Delancey JO Toglia MR Perucchini D. Internal and external anal sphincter anatomy as it relates to midline obstetric lacerations. Obstet Gynecol. (1997) 90:924–7. 10.1016/S0029-7844(97)00472-9 9397104

[6] Goldaber KG Wendel PJ McIntire DD Wendel GD Jr. Postpartum perineal morbidity after fourth-degree perineal repair. Am J Obstet Gynecol. (1993) 168:489–93. 10.1016/0002-9378(93)90478-2 8438915

[7] Venkatesh KS Ramanujam PS Larson DM Haywood MA. Anorectal complications of vaginal delivery. Dis Colon Rectum. (1989) 32:1039–41. 10.1007/BF02553877 2591278

[8] Handa VL Danielsen BH Gilbert WM. Obstetric anal sphincter lacerations. Obstet Gynecol. (2001) 98:225–30. 10.1097/00006250-200108000-00008 11506837

[9] Williams MK Chames MC. Risk factors for the breakdown of perineal laceration repair after vaginal delivery. Am J Obstet Gynecol. (2006) 195:755–9. 10.1016/j.ajog.2006.06.085 16949409

[10] Lewicky-Gaupp C Leader-Cramer A Johnson LL Kenton K Gossett DR. Wound complications after obstetric anal sphincter injuries. Obstet Gynecol. (2015) 125:1088–93. 10.1097/AOG.0000000000000833 25932836

[11] Stock L Basham E Gossett DR Lewicky-Gaupp C. Factors associated with wound complications in women with obstetric anal sphincter injuries (OASIS). Am J Obstet Gynecol. (2013) 208:327.e1–6. 10.1016/j.ajog.2012.12.025 23262251

[12] Barranger E Haddad B Paniel BJ. Fistula in ano as a rare complication of mediolateral episiotomy: report of three cases. Am J Obstet Gynecol. (2000) 182:733–4. 10.1067/mob.2000.102960 10739541

[13] Gordon PH. Rectovaginal fistula. Principles and Practice of Surgery for the Colon, Rectum, and Anus. 3rd ed. New York, NY: Informa Healthcare (2006). p. 333–52.

[14] Wiskind AK Thompson JD. Transverse transperineal repair of rectovaginal fistulas in the lower vagina. Am J Obstet Gynecol. (1992) 167:694–9. 10.1016/S0002-9378(11)91573-7 1530025

[15] Hull TL El-Gazzaz G Gurland B Church J Zutshi M. Surgeons should not hesitate to perform episioproctotomy for rectovaginal fistula secondary to cryptoglandular or obstetrical origin. Dis Colon Rectum. (2011) 54:54–9. 10.1097/01.dcr.0000388926.29548.36 21160314

